# Influence of Hydrogen and Ethanol Addition in Methanogen-Free Mixed Culture Syngas Fermentations in Trickle Bed Reactors

**DOI:** 10.3390/molecules29235653

**Published:** 2024-11-29

**Authors:** Cesar Quintela, Iulian-Gabriel Alexe, Yvonne Nygård, Lisbeth Olsson, Ioannis V. Skiadas, Hariklia N. Gavala

**Affiliations:** 1 Department of Chemical and Biochemical Engineering, Technical University of Denmark, 2800 Kongens Lyngby, Denmark; cquga@kt.dtu.dk (C.Q.); alexeiuliangabriel@gmail.com (I.-G.A.); ivsk@kt.dtu.dk (I.V.S.); 2 Division of Industrial Biotechnology, Department of Life Sciences, Chalmers University of Technology, SE 41296 Gothenburg, Sweden; yvonne.nygard@chalmers.se (Y.N.);

**Keywords:** syngas fermentation, electron donors, acetogenesis, chain elongation, solventogenesis

## Abstract

The use of mixed cultures in gas fermentations could reduce operating costs in the production of liquid chemicals such as alcohols or carboxylic acids. However, directing reducing equivalents towards the desired products presents the challenge of co-existing competing pathways. In this study, two trickle bed reactors were operated at acetogenic and chain elongating conditions to explore the fate of electron equivalents (ethanol, H_2_, and CO) and test pH oscillations as a strategy to target chain-elongated products. Hereby, the use of a H_2_-rich syngas increased gas conversion rates and the specificity towards acetic acid (86% of C-mol production, 9.0 g L_EBV_^−1^ day^−1^, with EBV referring to empty bed volume), while preliminary experiments with CO-rich syngas show promising results in increasing the ethanol production necessary to target chain-elongated products. On the other hand, ethanol supplementation hindered the endogenous ethanol production of the acetogenic culture but promoted butanol production (1.0 g L_EBV_^−1^ day^−1^) at high ethanol concentrations (9.6 g L^−1^) in the fresh media. Finally, pH oscillations improved chain elongation yields but negatively affected acetogenic growth, reducing production rates.

## 1. Introduction

Gas fermentation enables the production of fuels and commodity chemicals from industrial gaseous wastes, such as steel-milling CO-rich effluents, as well as from recalcitrant solid wastes through gasification and subsequent syngas biological conversion. This process could therefore play a key role in the decarbonization of the chemical industry [1]. It consists of the microbial conversion of the H_2_, CO, and CO_2_ present in the syngas or industrial off-gases towards products such as methane, ethanol, acetic acid, and longer chain alcohols or carboxylic acids [2]. To date, most commercial and pilot scale syngas fermentation processes are designed using pure microbial cultures, as they are easy to predict, operating conditions can be fine-tuned relatively easily, and genetic engineering approaches can be applied to optimise the conversion yields. Mixed culture fermentations, on the other hand, involve the use of rich microbial inocula, which result in more challenging optimization processes and may ultimately reach suboptimal conditions in comparison to pure culture fermentations. Nevertheless, the robustness and resilience of mixed cultures make them ideal hosts for industrial waste fermentations, as they can withstand substrate composition changes or toxic contaminants and can be run continuously without the need of sterility measures, significantly lowering the operating costs [3]. Specifically in the gas fermentations field, mixed cultures have also demonstrated a remarkable resilience and stability for biomethanation of real syngas and production of liquid products in trickle bed reactors (TBRs) [4,5,6].

To target liquid products, which are more energetically dense and exhibit higher market prices, methanogenesis can be suppressed in mixed culture fermentations by tuning the operating conditions [7,8], using chemical inhibitors, or heat pre-treating the microbial inoculum to remove methanogenic archaea [9]. In this way, the production can be steered towards liquid alcohols and carboxylic acids which hold higher market prices than methane. Moreover, conditions can be tuned to improve the selectivity of the culture towards acetic acid [10] or ethanol [11], which are the main products of acetogenic bacteria, or to further induce the chain elongation of the acetogenic products to longer carboxylic acids [5] or alcohols [8].

A key parameter affecting the efficiency of syngas fermentation processes is the syngas composition. Syngas is mainly composed of varying amounts of CO, H_2_, CO_2_, CH_4_, and N_2_ [12,13], depending on the source (e.g., steel-mill off-gases, wood gasification, etc.) and the gasification method. During syngas fermentation, acetogenic microbes fix the carbon present in CO_2_ and CO into acetic acid or ethanol through the Wood–Ljungdahl pathway using the reducing equivalents found in CO and H_2_. This conversion generates a surplus energy that the microbes can harness to grow [14]. CO is a more electronegative electron donor than H_2_, yielding more energy to the microbes and favouring alcohol production [15,16]. On the other hand, CO is also toxic to several microbial species present in gas-fermenting communities [17,18], and the fermentation of CO generates CO_2_, so H_2_ must be supplied to eliminate CO_2_ in the process effluent [19].

To target the production of longer acids or alcohols from syngas, the conditions must favour, on the one hand, acetogenesis to both acetic acid and ethanol, and on the other hand, the chain elongation reaction, which entails mesophilic and slightly acidic (pH 5.5–6.5) conditions [20,21]. In continuous processes, the production of ethanol in the acetogenic step is usually limiting for the chain elongation reaction leading to a surplus of acetic acid in the reactors [5,21]. To circumvent this limitation, Fernández-Blanco et al. tested an exogenous ethanol addition to stirred tank reactors operated in batch mode to convert syngas to caproic acid, reaching high caproic acid titers (8.2 g L^−1^) [22]. However, this strategy could pose more challenges in mixed culture studies, where the ethanol added could be consumed by competing routes [23].

Despite the success of electron donor addition in enhancing the production of longer-chain acids and alcohols from syngas in pure cultures, few studies have tested these strategies in mixed cultures. In this study, two TBRs with pH and temperature control performing syngas fermentation [5] were used to analyse the effect of different electron donors (mainly H_2_ and ethanol) on the performance of methanogen-free syngas fermenting communities. Additionally, a strategy based on pH cycles was tested with the hypothesis that it could improve the ethanol production rates of the acetogenic culture as an alternative to exogenous ethanol addition. The aim of these experiments was to enhance the TBRs’ CO_2_ consumption and chain elongation yields, and to investigate how these strategies would impact syngas fermentation yields and consumption rates when using mixed cultures.

## 2. Results and Discussion

### 2.1. Experimental Overview

At the start of the study, two TBRs were running steadily converting syngas to a mixture of acetic, butyric, and caproic acid. The starting conditions were: 37 °C, pH 6, 3 days liquid hydraulic retention time (HRT), 40 min gaseous empty bed residence time, and a syngas composition of 45% H_2_, 25% CO_2_, 20% CO, and 10% N_2_. The reactors had previously been operated for 12 months on syngas at different pH values as described in Quintela et al. [5]. After achieving steady-states at the starting conditions, referred to throughout this study as the original steady-states, the conditions in the TBR were changed to test different syngas mixtures in TBR1, and exogenous ethanol addition and pH oscillations in TBR2 (Table 1). After each test, the operating conditions of the TBR were returned to the starting conditions, to reach control steady-states. Subsequently, the steady-states reached at the test conditions were compared to the previous original or control steady-state reached in the TBR. Additionally, the differences among all original and control steady-states reached were assessed to evaluate the evolution of the TBRs communities through the study. The CO-rich test did not reach a steady-state at the time of the completion of the study, but the preliminary results obtained are presented in the Supplementary Material. 

**Figure 1:**
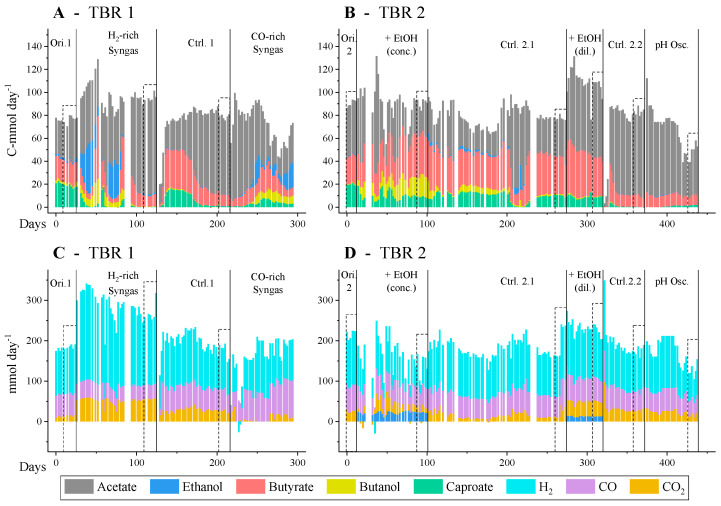
Production (**A**,**B**) and consumption (**C**,**D**) profile in the two TBRs. The different conditions tested are separated in all graphs by vertical lines and labelled at the top of the graph. Additionally, the steady-state periods are highlighted in dashed boxes.

The two TBR (TBR1 and TBR2) were run continuously for 300 and 450 days, respectively; the profiles of CO, H_2_, and CO_2_ consumption and product generation are shown in Figure 1. Regardless of the changes on the consumption and production profile during the transition periods before the steady-states were reached, the reactor’s performance was evaluated solely based on steady-state operation. The changes happening during transition periods could be due to possible disruptions of the normal operation of the reactors. At the original conditions, the reactors produced a mixture of carboxylic acids from syngas, namely acetic acid (46 ± 2% of the C-mol in the products), butyric acid (26 ± 1%), and caproic acid (23 ± 1%). On average, 121, 59, and 20 mmol day^−1^ of H_2_, CO, and CO_2_, respectively, were consumed in this period. This means 64% of the H_2_ and 70% of the CO fed were consumed, while only 19% of the CO_2_ was converted. The measured CO_2_ conversion depends on both the production (through CO fermentation) and consumption of CO_2_. Nevertheless, the fact that the overall CO_2_ conversion was low at the original conditions can be easily explained by the relationship between carbon moles and electron moles present in the substrates and in the products. At these conditions, the syngas mix fed presented an electron mol to carbon mol ratio (e:C ratio) of 2.9. On the other hand, the e:C of the carboxylic acids present is 4 for acetic acid, 5 for butyric acid, and 5.3 for caproic acid. Thus, the research question raised was whether supplementation with additional electron donors would improve the consumption rates of syngas, as it could enable the conversion of more CO_2_ into ethanol and acetic acid in the acetogenic step. Moreover, whether a more reducing substrate could increase the ethanol:acetic acid ratio reaching the chain elongation step, thus improving the yield of chain-elongated acids. 

### 2.2. Gaseous Electron Donors

**Figure 2:**
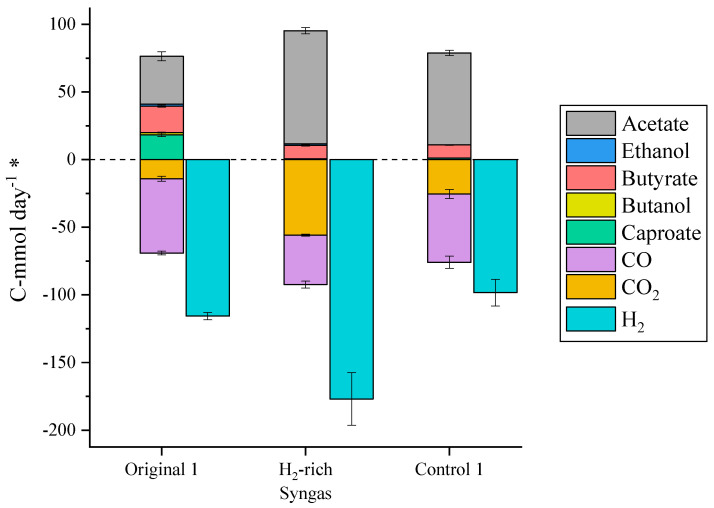
Production (positive numbers) and consumption (negative numbers) of the main extracellular metabolites in each of the steady-states reached in TBR1. * All metabolites in the graph are shown in C-mmol day^−1^ except for H_2_, which is expressed in mmol day^−1^.

The supplementation of the syngas with H_2_ to reach an e:C of 6 (Table 1) drastically increased the absolute CO_2_ consumption rates (Figure 2), even though less CO_2_ was fed to the system. The percentage of CO_2_ consumed in TBR1 increased from 13 to 90%, while the percentage of CO consumed increased from 65 to 73%. In contrast, the percentage of H_2_ remained constant (61–62%). Regarding the product profile, the change in the syngas mix had a negative effect in the production of elongated acids and increased the specificity of the process towards acetic acid, which reached 86% of the C-mol production. Together with the higher consumption rates observed, it also resulted in high acetic acid titers (18.6 g L^−1^) and productivities (9.0 g L_EBV_^−1^ day^−1^). Returning the reactor to control conditions did not restore the original production and consumption performance (Figure 2), implying either a change in the microbial community inside the TBR or that more time was needed for a complete recovery of the original production. 

A possible explanation for the product profile obtained under H_2_-rich syngas is that the supplementation of syngas with additional H_2_ did not only result in a higher e:C ratio, but also in the reduction of the electron equivalents fed in the form of CO. CO has a lower reduction potential than H_2_ and, thus, can directly reduce ferredoxin and generate ATP through chemiosmotic phosphorylation [15]. Therefore, CO consumption favours the production of alcohols over acids as they entail higher consumption of electron equivalents and, thus, ATP generation per mol of product generated [16]. This agrees with the obtained results, as the supplementation with H_2_ and reduction of the CO fed reduced the yield of chain-elongated products, probably because of a reduction in the ethanol production as an intermediary in the acetogenic step of the process. Additionally, a preliminary experiment performed in TBR1 showed an increase in solventogenic activity when the syngas composition was changed to a 67% H_2_, 33% CO mix (Figure S1). Therefore, while H_2_-rich syngas mixes can improve the specificity of syngas fermentation processes towards acetic acid production, the use of more CO-rich syngas mixes shows potential for the production of elongated acids and deserves further investigation.

### 2.3. Exogenous Ethanol Addition

Acetic acid is usually the most abundant product in continuous syngas fermentation processes targeting the production of chain-elongated acids [5,20,24]. To elongate acetic acid and the resulting elongated carboxylic acids, more ethanol is needed to act as an electron donor. Fernández-Blanco [22] proved that the exogenous addition of ethanol to a co-culture fermenting syngas could improve the yields of chain-elongated acids. In this study, ethanol was supplemented to the liquid media at two different concentrations to study the impact it would have in the acetogenic and chain elongating steps of the process.

**Figure 3:**
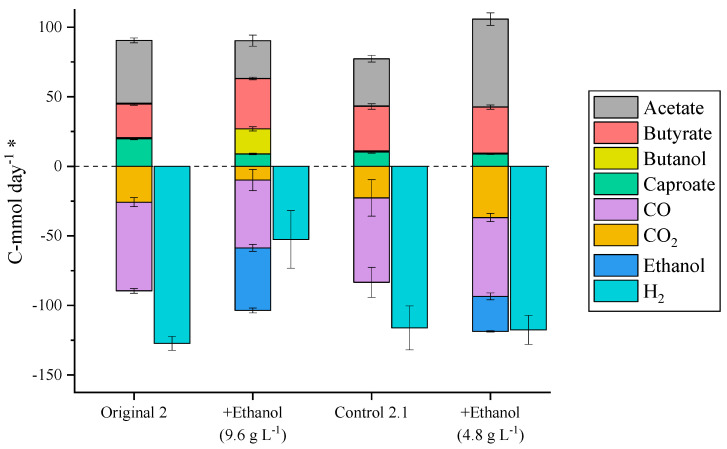
Production (positive numbers) and consumption (negative numbers) of the main extracellular metabolites in the first four steady-states reached in TBR2. * All metabolites in the graph are shown in C-mmol day^−1^ except for H_2_, which is expressed in mmol day^−1^.

The supplementation of the media with 9.6 g L^−1^ ethanol had a negative impact in the acetogenic step of the process, reducing the consumption of CO_2_, CO, and H_2_ by 62, 23, and 59%, respectively (Figure 3). On the other hand, the addition of ethanol promoted the elongation of acetic acid to butyric acid, which reached the maximum concentration (6.0 g L^−1^) and productivity (2.5 g L_EBV_^−1^ day^−1^) obtained in this study. From butyric acid though, instead of promoting the elongation to caproic acid, as it was seen in co-culture studies [22], ethanol addition resulted in electron equivalents being directed towards the production of butanol (2.5 g L^−1^, 1.0 g L_EBV_^−1^ day^−1^). In this experiment, 19% of the ethanol fed left the reactor unconverted, and ethanol concentrations inside the reactor remained around 1.8 g L^−1^ despite acetic acid also being present at a concentration of 6.1 g L^−1^; this might be due to exogenous ethanol addition hindering endogenous ethanol generation while remaining ethanol present in the fermentation broth triggered the high solventogenesis rates observed. More research is needed before drawing solid conclusions on the reasons for this response.

Subsequently, the reactor was set back to control conditions. After a baseline steady-state was obtained, ethanol supplementation was tested at a concentration of 4.8 g L^−1^, to decrease the ethanol concentration inside the reactor and prevent butanol formation. In this case, 97% of the ethanol fed was converted to products. In contrast to the 9.6 g L^−1^ ethanol supplementation experiment, the consumption of H_2_ and CO remained at similar levels, while the CO_2_ consumption increased by 62% (Figure 3). Furthermore, acetic acid production increased, and butyric and caproic acid production remained constant. Taking into account the chain elongation stoichiometries (see Section 3.3), around 13.5 mmol day^−1^ ethanol were necessary in control 2.1 and in the 4.8 g L^−1^ supplementation experiment to produce the butyric and caproic acid present. In control 2.1, this ethanol was produced by acetogenic bacteria, while during the 4.8 g L^−1^ supplementation experiment, 12.6 mmol day^−1^ of ethanol was consumed from the ethanol provided exogenously and, therefore, only 0.9 mmol day^−1^ ethanol was produced by acetogenic bacteria. This implies that the addition of exogenous ethanol diverged the acetogenic metabolism towards the production of acetic acid and supports the assumption that exogenous ethanol addition hinders endogenous ethanol generation. Additionally, the change in the acetogenic production profile did not affect the consumption of electron donors (H_2_ and CO), which remained constant, but resulted in higher CO_2_ consumption rates as acetic acid production requires less electron equivalents per carbon consumed than ethanol production. 

### 2.4. pH Oscillations to Improve Endogenous Ethanol Production

**Figure 4:**
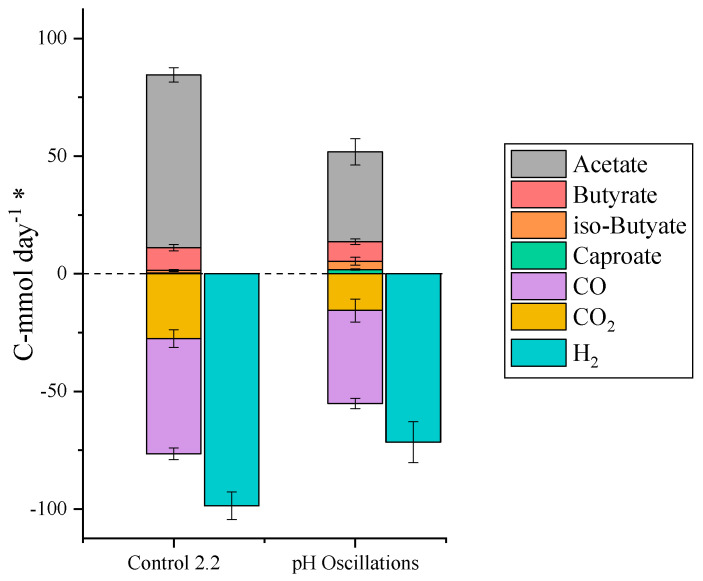
Production (positive numbers) and consumption (negative numbers) of the main extracellular metabolites in the last two steady-states reached in TBR2. * All metabolites in the graph are shown in C-mmol day^−1^ except for H_2_, which is expressed in mmol day^−1^.

The control 2.2 set in TBR2 after the 4.8 g L^−1^ ethanol supplementation showed a very high specificity towards acetic acid production (86% of C-mol produced). Most probably the suppression of endogenous ethanol production, induced by the exogenous addition of ethanol, was not recovered after stopping the ethanol supplementation. To restore the production of ethanol, a strategy based on pH cycles, i.e., 24 h cycles comprised of 18 h of natural acidification and 6 h of pH control at pH 6, was tested. The use of pH cycles has proven successful to improve ethanol production yields from syngas in pure cultures [25], but it had not been tested in mixed cultures before. Hereby, the pH cycles did improve the yields of butyric and caproic acid (16 and 3% of C-mol production, respectively, vs. 11 and 0% in control 2.2), but decreased the gas consumption and therefore the overall productivity of the process (Figure 4). An explanation could be that the pH cycles disturbed the acetogenic growth, as the suspended biomass concentration decreased significantly during this study (Table S1, Supplementary Material). Additionally, the production of iso-butyric acid was the highest in this study, reaching 0.6 g L^−1^ and implying a positive impact of pH cycles in the production of branched carboxylic acids.

### 2.5. Evolution of the Microbial Community Through the Study

**Figure 5:**
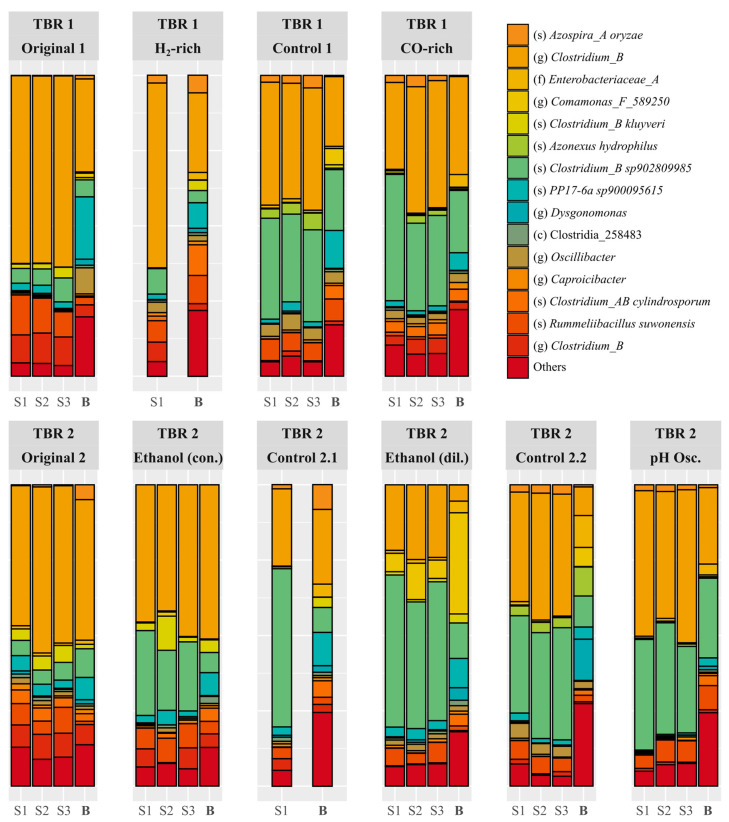
Relative abundance of the main ASVs identified in biofilm (**B**) and suspended growth (S1, S2, S3) samples in the steady-states reached in the two TBRs. The deepest taxonomic level known for each ASV is indicated in the legend as class (c), family (f), genus (g), and species (s). The species was only given for confidence values above 99%.

The H_2_-rich syngas and 4.8 g L^−1^ ethanol supplementation conditions resulted in a decline of ethanol production in the acetogenic step of the process, which was not recovered when the reactors were set back to control conditions (Figure 2 and Figure 3). Samples of suspended and biofilm biomass were taken at each of the steady-states reached in this study, to compare the evolution of the microbial community (using 16S rDNA analysis) with the performance of the reactor at the different operational conditions. The analysis of the community at genus level did not show much variation through the study, with *Clostridium* representing more than 75% of the community in all samples (Figure S2, Supplementary Material). In 16S rDNA analyses, the distinct ASVs obtained are usually merged at genus level because the 16S rRNA gene fragment sequenced is usually not long enough to identify most microbial species with confidence. However, in mixed culture gas fermentation studies, the *Clostridium* genus comprises many different acetogenic, chain elongating, and even sulphate reducing species [26], with different growth, consumption, and production profiles and, thus, analysing the microbial community at genus level can, in some cases, offer little additional information. To overcome this challenge, the relative abundance of the most abundant ASVs across the different conditions was analysed before merging them at genus level (Figure 5). In this way, a change in the ASV or microbial strain responsible for the conversion could be determined even if the particular species change within the genus cannot be identified. Although the suspended growth samples from the original steady-states are dominated by the *Clostridium* ASV labelled as “Clostridium_B” (Figure 5, orange), the control steady-states samples, run at the same conditions as the original ones, exhibit similar abundances of the “Clostridium_B” ASV and another *Clostridium* ASV identified as “Clostridium_B sp902809985” (Figure 5, green). Based on the above, supplementation of additional electron donors (i.e., H_2_ and ethanol) altered the acetogenic *Clostridium* community, leading to a decline in ethanol production at the acetogenic step of the process, which in turn reduced the production of chain-elongated acids, i.e., butyric and caproic acids.

## 3. Materials and Methods

### 3.1. Growth Medium

The reactors were supplied with a modified basal anaerobic (BA) medium to provide the culture with essential nutrients for microbial growth. Following the method outlined by Quintela et al. [5], the following stock solutions were prepared: (1) macronutrients (NH_4_Cl, 100 g L^−1^; NaCl, g L^−1^; MgCl_2_•6H_2_O, 10 g L^−1^; CaCl_2_•2H_2_O, 5 g L^−1^), (2) dipotassium hydrogen phosphate solution (K_2_HPO_4_•3H_2_O, 200 g L^−1^), (3) sodium sulphate solution (Na_2_SO_4_, 100 g L^−1^), (4) sodium sulphide solution (Na_2_S, 24.975 g L^−1^), (5) vitamin solution (biotin, 10 mg L^−1^; folic acid, 10 mg L^−1^; pyridoxine HCl, 50 mg L^−1^; riboflavin HCl, 25 mg L^−1^; thiamine HCl, 25 mg L^−1^; cyanocobalamin, 0.5 mg L^−1^; nicotinic acid, 25 mg L^−1^; p-aminobenzoic acid, 25 mg L^−1^; lipoic acid, 25 mg L^−1^; d-pantothenic acid hemicalcium salt, 25 mg L^−1^), and (6) modified ATCC 1754 trace metal (micronutrients) solution (nitrilotriacetic acid, 2000 mg L^−1^; MnSO_4_•H_2_O, 1119 mg L^−1^; Fe(SO_4_)_2_(NH_4_)_2_•6H_2_O, 800 mg L^−1^; CoCl_2_•6H_2_O, 200 mg L^−1^; ZnSO_4_•7H_2_O, 200 mg L^−1^; CuCl_2_•2H_2_O, 20 mg L^−1^; NiCl_2_•6H_2_O, 20 mg L^−1^; Na_2_MoO_4_•2H_2_O, 20 mg L^−1^; Na_2_SeO_3_•5H_2_O, 27 mg L^−1^; Na_2_WO_4_•2H_2_O, 25 mg L^−1^; H_3_BO_3_, 10 mg L^−1^; AlCl_3_, 10 mg L^−1^).

To prepare the modified BA medium, deionised water was combined with the following quantities of stock solutions: macronutrients, 20 mL L^−1^; dipotassium hydrogen phosphate solution, 5 mL L^−1^; sodium sulphate solution, 10 mL L^−1^; sodium sulphide solution, 0.2 mL L^−1^; vitamin solution, 10 mL L^−1^; and trace metal solution, 10 mL L^−1^. Yeast extract was also supplemented to the media to a final concentration of 0.5 g L^−1^, leading to a final pH of 7.4.

### 3.2. Experimental Set-Up

The configuration of the TBRs used was described in detail by Quintela et al. [5]. Briefly, two TBRs were operated continuously throughout this study, each consisting of a column filled with polypropylene/polyethylene packing material, where the gas conversion to liquid products takes place, and a liquid reservoir. The empty bed volume (EBV) of the TBR column was 300 mL, while the reservoir liquid volume was 400 mL. The liquid was continuously recirculated from the reservoir to the column using a peristaltic pump, at a liquid recirculation rate of 81 L L_EBV_^−1^ day^−1^. The gas flow was controlled with gas flow controllers and the liquid flow was controlled with a peristaltic pump. The pH was controlled using a pH electrode placed in the liquid reservoir, and pH transmitter controlling pumps connected to acid (HCl 0.5 M) and base (KOH 5 M) solutions. Additionally, the temperature was kept constant with a water bath connected to the water jackets of the TBR column and reservoir.

### 3.3. Analytical Techniques and Stoichiometric Calculations

The gas outflow composition was analysed using a gas chromatograph (8610C, SRI Instruments, Bad Honnef, Germany) equipped with a thermal conductivity detector and two packed columns (6′ × 1/8″ Molsieve 13× column and 6′ × 1/8″ silica gel column) connected in series through a rotating valve. The columns were maintained at 65 °C for 3 min, followed by a 10 °C min^−1^ ramp till 95 °C, and a 24 °C min^−1^ ramp reaching 140 °C. Gas samples of 50 μL were collected and injected with a gas-tight syringe (model 1750SL, Hamilton, Giarmata, Romania) [11]. To determine volatile fatty acids and alcohols, a high-performance liquid chromatograph (Shimadzu, Ballerup, Danmark) with a refractive index detector and an Aminex HPX-87H column (Bio-Rad, Copenhagen, Denmark) was employed, maintained at 60 °C. Finally, 12 mM H_2_SO_4_ was used as eluent at a flow rate of 0.6 mL min^−1^.

The chain elongation stoichiometries used to estimate the ethanol produced in the acetogenic step of the process were the same as those used by Quintela et al. [27] and are based on the chain elongation stoichiometries reported by Spirito et al. [28].

$$6\;C_{2}H_{6}O+4\;C_{2}H_{3}O_{2}^{-}\to 5\;C_{4}H_{7}O_{2}^{-}+H^{+}+4\;H_{2}O+2\;H_{2}\;6\;C_{2}H_{6}O+5\;C_{4}H_{7}O_{2}^{-}\to 5\;C_{6}H_{11}O_{2}^{-}+C_{2}H_{3}O_{2}^{-}+H^{+}+4\;H_{2}O+2\;H_{2}$$

### 3.4. Community Analysis

For the analysis of the suspended communities, plastic carriers carrying biofilm were collected from the upper half of the reactor. Subsequently, the biomass was washed out of the carrier by shaking it vigorously into fresh liquid media. Additionally, 10 mL liquid samples were taken from the TBRs. All samples were then centrifuged to discard the supernatant, and frozen at −20 °C for simultaneous processing at the end of the experiment. The DNA was extracted using a DNeasy Blood and Tissue Kit (Qiagen, Hvidovre, Denmark), and submitted to Macrogen Inc. (Seoul, Republic of Korea) for 16S amplicon library preparation and sequencing using Illumina Miseq (300 bp paired-end sequencing). The libraries were constructed following the 16S Metagenomic Sequencing Library Preparation Protocol (Part #15044223, Rev. B) using Herculase II Fusion DNA Polymerase Nextera XT Index Kit V2. Regions V4-V5 of 16S rRNA gene were amplified with primers 515F (5′-GTGYCAGCMGCCGCGGTAA-3′) and 926R (5′-CCGYCAATTYMTTTRAGTTT-3′) [29]. Raw reads were merged, quality-filtered, and denoised, using the DADA2 algorithm within the Qiime2 pipeline, to obtain amplicon sequence variants (ASVs) [30]. Taxonomic assignment was then performed using a classify-sklearn algorithm, along with a classifier trained on the Greengenes2 (2022.10) database. Further downstream analysis was conducted using the phyloseq and ggpubr packages in R (version 4.2.1).

## 4. Conclusions

In this study, increasing the H_2_ percentage in the syngas enhanced CO_2_ conversion and reduced ethanol production during the acetogenic step, directing most of the electrons towards acetic acid, which achieved high productivities (9.0 g L_EBV_^−1^ day^−1^). This shift could be attributed to the reduced CO percentage in the syngas mix, as preliminary experiments using a 2:1 H_2_:CO mix showed increased endogenous ethanol production. Ethanol supplementation (9.6 g L^−1^) slowed gas conversion rates down and triggered a solventogenic metabolic switch that led to butanol production (1.0 g L_EBV_^−1^ day^−1^), most likely due to the excess unconverted ethanol in the reactor. In contrast, lower ethanol supplementation (4.8 g L^−1^) increased CO_2_ conversion rates substantially, supressed endogenous ethanol production, and steered the acetogenic metabolism towards acetic acid. The cyclic-pH strategy improved the yields of chain-elongated acids slightly but reduced the growth and production rates of the acetogenic community significantly. The 16S rRNA analysis showed that the decline in endogenous ethanol production by the microbial community coincided with a change in the *Clostridium* species dominating the culture. This change was not reverted throughout the study and implies that either more time was needed for a complete recovery of the original microbial consortium or re-inoculation may sometimes be necessary in order to restore chain elongating activity. More investigation is needed for drawing solid conclusions on this. Using syngas mixture with high e:C ratio and CO percentages, such as the 2:1 H_2_:CO mix, showed promising potential for increasing the yields of chain-elongated products from syngas by mixed cultures.

## Figures and Tables

**Table 1:** Conditions tested in TBR1 and TBR2 throughout the study.

Reactor	Condition	Description	Days
TBR 1	Original 1	Starting conditions (45% H_2_, 25% CO_2_, 20% CO, 10% N_2_)	0–24
TBR 1	H_2_-rich Syngas	Syngas composition changed to 68% H_2_, 15% CO_2_, 12% CO, 6% N_2_	24–124
TBR 1	Control 1	Starting conditions	124–215
TBR 1	CO-rich syngas	Syngas composition changed to 67% H_2_, 33% CO	215–294
TBR 2	Original 2	Starting conditions	0–11
TBR 2	+Ethanol (9.6 g L^−1^)	Ethanol supplementation in liquid media (9.6 g L^−1^)	11–100
TBR 2	Control 2.1	Starting conditions	100–273
TBR 2	+Ethanol (4.8 g L^−1^)	Ethanol supplementation in liquid media (4.8 g L^−1^)	273–319
TBR 2	Control 2.2	Starting conditions	319–371
TBR 2	pH Oscillations	24 h cycles comprised of: 18 h uncontrolled pH, 6 h of pH 6 setting	371–438

## Data Availability

Data reported in this study are available upon request.

## Supplementary Materials

The following supporting information can be downloaded at: https://www.mdpi.com/article/10.3390/molecules29235653/s1, Figure S1: Production (positive numbers) and consumption (negative numbers) of the main extracellular metabolites in each of the steady-states reached in TBR1, compared to the averaged last datapoints of the CO-rich syngas preliminary test; Table S1: Optical density measured at 600 nm (OD_600_) and calculated suspended cells concentration; Figure S2: Relative abundance of the main genera identified in biofilm (B) and suspended growth (S1, S2, S3) samples in the steady-states reached in the two TBR.
