# Supplementary material for: Influence of Hydrogen and Ethanol Addition in Methanogen-Free Mixed Culture Syngas Fermentations in Trickle Bed Reactors
Source: Molecules. 2024 Nov 29;29(23):5653. doi: 10.3390/molecules29235653 (PMC11643839; doi:10.3390/molecules29235653)
Supplement: Supplementary file 1 [file molecules-29-05653-s001.zip › molecules-3298252-supplementary.pdf]

molecules

MDPI

Supplementary material for Article

Influence of Hydrogen and Ethanol Addition in Methanogen-Free Mixed Culture Syngas Fermentations in Trickle Bed Reactors

Cesar Quintela [1], Iulian-Gabriel Alexe [1], Yvonne Nygård [2], Lisbeth Olsson [2], Ioannis V. Skiadas [1] and Hariklia N. Gavala [1,*]

[1] Department of Chemical and Biochemical Engineering, Technical University of Denmark, 2800 Kongens Lyngby, Denmark; cquga@kt.dtu.dk (C.Q.); alexeiuliangabriel@gmail.com (I.-G.A.); ivsk@kt.dtu.dk (I.V.S.)
[2] Division of Industrial Biotechnology, Department of Life Sciences, Chalmers University of Technology, SE 41296 Gothenburg, Sweden; yvonne.nygard@chalmers.se (Y.N.)
* Correspondence: hnga@kt.dtu.dk or hari_gavala@yahoo.com

S1. CO preliminary experiment

After a steady-state was reached for the Control 1 condition in TBR1, the syngas was changed to a 2:1 H_2:CO gas mix, so that the same e:C ratio as in the H_2-rich syngas, but in this case with a high CO percentage too. The rationale for this is that higher CO partial pressures could enhance the production of ethanol by the acetogenic community and thus increase the production of chain-elongated molecules. Although a steady state was not reached for the CO-rich syngas condition before the reactors had to be shut down, the averaged last datapoints of the test (the whole period can be seen in Figure 1 A and C) is averaged and compared to previous test in Figure S1.

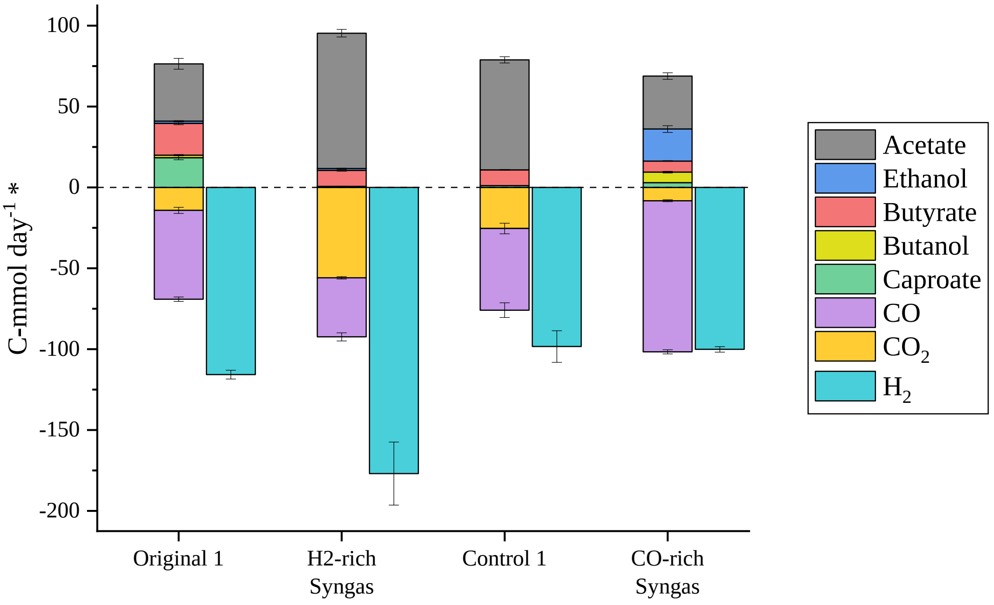

Figure S1. Production (positive numbers) and consumption (negative numbers) of the main extracellular metabolites in each of the steady states reached in TBR1, compared to the averaged last datapoints of the CO-rich syngas preliminary test. *All metabolites in the graph are shown in C-mmol day^{-1} except for H_2, which is expressed in mmol day^{-1}.

S2. OD_{600}

The OD_{600} was measured in each of the steady states reached in the TBRs, and the suspended cell concentrations was calculated. However, TBR performance is based on biofilm formation and therefore one cannot directly correlate the suspended cells with the reactor efficiency. Nevertheless, a sharp increase or decrease of the suspended cells could be a sign of disturbance and this is what was noticed in the steady state reached at the pH oscillation experiment.

Table S1. Optical density measured at 600 nanometers (OD_{600}) and calculated suspended cells concentration. The cell concentration (g L^{-1}) was calculated according to the calibration performed in [1] for the same reactors. Conditions tested in TBR1 and TBR2 throughout the study.

TBR	Condition	OD_{600}	Cells concentration (g L^{-1})
1	Original 1	1.55 ± 0.08	0.48 ± 0.02
1	H_2-rich Syngas	1.31 ± 0.10	0.41 ± 0.03
1	Control 1	1.64 ± 0.11	0.51 ± 0.03
1	CO-rich syngas	1.18 ± 0.16	0.38 ± 0.05
2	Original 2	1.76 ± 0.18	0.54 ± 0.05
2	+ Ethanol (con.)	1.66 ± 0.16	0.51 ± 0.05
2	Control 2.1	2.12 ± 0.32	0.65 ± 0.09
2	+ Ethanol (dil.)	1.98 ± 0.11	0.61 ± 0.03
2	Control 2.2	1.88 ± 0.29	0.58 ± 0.08
2	pH Oscillations	0.78 ± 0.03	0.26 ± 0.01

S3. 16S rRNA community analysis

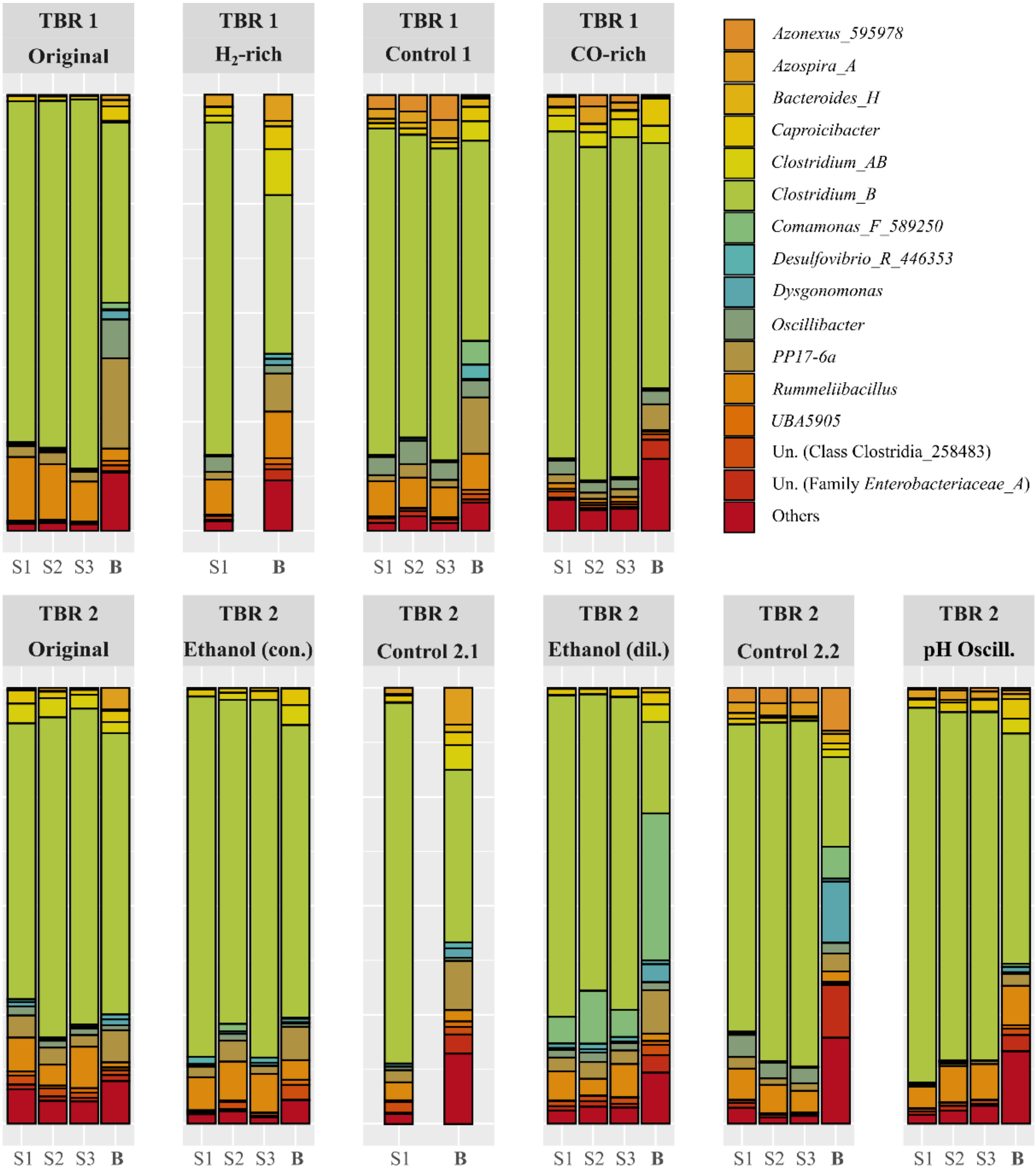

Figure S2. Relative abundance of the main genera identified in biofilm (B) and suspended growth (S1, S2, S3) samples in the steady states reached in the two TBR.

References for Supplementary Material

1. Quintela, C.; Grimalt-Alemany, A.; Modin, O.; Nygård, Y.; Olsson, L.; Skiadas, I. V.; Gavala, H.N. Effect of PH in Syngas Conversion to C4 & C6 Acids in Mixed-Culture Trickle Bed Reactors. *Biomass Bioenergy* 2024, *187*, 107292, doi:10.1016/j.biombioe.2024.107292.
